# Strain specific transcriptional response in *Mycobacterium tuberculosis *infected macrophages

**DOI:** 10.1186/1478-811X-10-2

**Published:** 2012-01-26

**Authors:** Mi-Sun Koo, Selvakumar Subbian, Gilla Kaplan

**Affiliations:** 1 Laboratory of Mycobacterial Immunity and Pathogenesis, The Public Health Research Institute (PHRI) at the University of Medicine and Dentistry of New Jersey (UNDNJ), 225 Warren Street, Newark, New Jersey 07103, USA

**Keywords:** Tuberculosis, Bone marrow-derived macrophage, Gene expression, Global transcriptome, *Mycobacterium tuberculosis*, Macrophage activation pathway, Host-pathogen interaction, Immune response, Lipid metabolism, Intracellular stress response

## Abstract

**Background:**

Tuberculosis (TB), a bacterial infection caused by *Mycobacterium tuberculosis *(*Mtb) *remains a significant health problem worldwide with a third of the world population infected and nearly nine million new cases claiming 1.1 million deaths every year. The outcome following infection by *Mtb *is determined by a complex and dynamic host-pathogen interaction in which the phenotype of the pathogen and the immune status of the host play a role. However, the molecular mechanism by which *Mtb *strains induce different responses during intracellular infection of the host macrophage is not fully understood. To explore the early molecular events triggered upon *Mtb *infection of macrophages, we studied the transcriptional responses of murine bone marrow-derived macrophages (BMM) to infection with two clinical *Mtb *strains, CDC1551 and HN878. These strains have previously been shown to differ in their virulence/immunogenicity in the mouse and rabbit models of pulmonary TB.

**Results:**

In spite of similar intracellular growth rates, we observed that compared to HN878, infection by CDC1551 of BMM was associated with an increased global transcriptome, up-regulation of a specific early (6 hours) immune response network and significantly elevated nitric oxide production. In contrast, at 24 hours post-infection of BMM by HN878, more host genes involved in lipid metabolism, including cholesterol metabolism and prostaglandin synthesis were up-regulated, compared to infection with CDC1551.

In association with the differences in the macrophage responses to infection with the 2 *Mtb *strains, intracellular CDC1551 expressed higher levels of stress response genes than did HN878.

**Conclusions:**

In association with the early and more robust macrophage activation, intracellular CDC1551 cells were exposed to a higher level of stress leading to increased up-regulation of the bacterial stress response genes. In contrast, sub-optimal activation of macrophages and induction of a dysregulated host cell lipid metabolism favored a less stressful intracellular environment for HN878. Our findings suggest that the ability of CDC1551 and HN878 to differentially activate macrophages during infection probably determines their ability to either resist host cell immunity and progress to active disease or to succumb to the host protective responses and be driven into a non-replicating latent state in rabbit lungs.

## Background

Tuberculosis (TB) remains one of the leading infectious diseases worldwide. The outcome of infection with *Mycobacterium tuberculosis (Mtb) *is determined by a complex and dynamic interaction between the host immune system and properties of the pathogen. Exploring the mechanisms of mycobacteria-phagocyte interactions is crucial to understanding the pathogenesis of TB. Aerosolized *Mtb*, once inhaled into the lung and internalized by alveolar macrophages can induce phagocyte activation leading to control of bacillary growth and establishment of a state of non-replicating persistence [1-3]. Alternatively, as seen in 10% of the infected immune competent humans, the bacilli can survive the hostile environment of the phagosome through interference with a range of cellular processes, including subversion of activation of bactericidal responses and blocking phagosome acidification and fusion with lysosomes [4,5]. Thus, when the activation of host immunity is optimal, infection by *Mtb *is controlled and lung pathology is minimal [6]. However, when macrophage activation is suboptimal, the bacilli continue to grow inside macrophages, additional cells are recruited from the circulation to the site of infection in the lungs and granulomas are formed [7-9].

The host immune responses to *Mtb *clinical strains CDC1551 and HN878 have previously been characterized *in vitro *and *in vivo *[10-14]. While infection by CDC1551 elicited an early and more vigorous pro-inflammatory cytokine response in human monocytes and stronger T cell activation in the lungs of infected mice, infection by HN878 induced reduced production by monocytes of Th1-type cytokines and diminished protective immunity in infected mice [10]. Moreover, in rabbit lungs, infection by HN878 was not controlled, resulting in progressive cavitary disease [6,11], while growth of infecting CDC1551 was limited by the immune response and cultivable bacilli were fully cleared from the lungs by 3-4 months post infection [12]. The hyper-virulent phenotype of *Mtb *HN878 has been shown to be associated with the production of phenolic glycolipid (PGL), one of a number of lipid components of the mycobacterial cell wall that may subvert Th1-type host protective immunity [13-16]. However, the early cellular and molecular events occurring in infected macrophages that are associated with differential outcome following infection with HN878 versus CDC1551 are not fully understood.

In the present study, using genome-wide transcriptome analysis, we examined the early changes in gene expression in bone marrow-derived macrophages (BMM) following infection with either HN878 or CDC1551. In addition, we analyzed the transcriptional response of selected genes of the intracellular bacteria, including those involved in the stress response and lipid metabolism. Our findings suggest that infection by CDC1551 of BMM elicits a rapid and strong immune activation program as early as 6 hours, compared to infection by HN878. In contrast, while the activation of BMM upon infection by HN878 was suboptimal at earlier time points, the response was more prolonged and included the induction of genes involved in host cell lipid and small molecule metabolism. Moreover, compared to HN878, intracellular CDC1551 showed a significantly increased level of expression of bacillary stress response genes involved in hypoxia/anaerobiosis. Dysregulation of macrophage lipid metabolism has been reported in surgically removed lung specimens from TB patients, with severe caseating granulomatous disease [17].

## Results

### Global changes in macrophage transcriptional response to infection by *Mtb*

**Figure 1:**
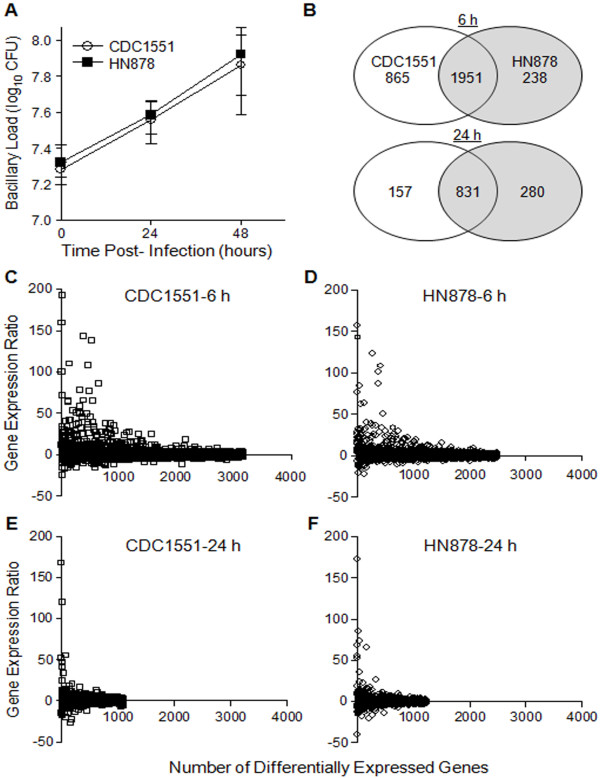
**Intracellular growth of CDC1551 and HN878 and global transcriptome of BMM during infection**. (A) Growth of CDC1551 and HN878 in macrophages up to 48 hpi. The cells were infected with a multiplicity of infection (MOI) of 5 (bacteria per phagocyte) for 3 hours. Equal numbers of BMM were seeded at the start of infection with CDC1551 and HN878. At 3 (T = 0), 24 and 48 hpi, cells were lysed and serial dilutions of the homogenates were plated to determine the CFU. The viability of the infected cells, measured by trypan blue exclusion, did not change significantly till the end of the experiment (48 hours). (B) Venn diagram of microarray data showing macrophage genes significantly differentially expressed (at least 2 fold change in expression and *P *≤ 0.05) by CDC1551 and HN878 at 6 and 24 hpi. (C and D) Transcriptional profile of individual host genes differentially expressed by CDC1551 (C) and HN878 (D) infection at 6 hpi. (E and F) Total number of differentially expressed macrophage genes by CDC1551 (E) and HN878 (F) infection at 24 hpi. The data presented in (C-F) is derived from microarray analysis of differentially expressed macrophage genes during infection by *Mtb *. Each spot represents a gene and all genes are ranked based on their relative expression ratio (*Mtb*-infected vs. uninfected cells). The microarray results were obtained from three independent RNA samples per experimental group (infected or uninfected).

Mouse BMM were infected with either CDC1551 or HN878 for up to 48 hours and the intracellular growth of the two *Mtb *strains was evaluated by the colony forming units (CFU) assay. Similar to our previous reports, there was no significant difference in the intracellular growth and total bacillary load between CDC1551 and HN878 during infection of BMM (Figure 1A) [12,16]. To study the changes in global transcriptome of host cells during infection by *Mtb*, levels of expression of BMM genes at 6 and 24 hours post-infection (hpi) were compared to the corresponding transcriptome from uninfected cells. Based on our selection criteria (at least 2 fold change in expression and *P *≤ 0.05), among the 28,853 genes represented in the microarray, expression of only 10.6% (3,054 genes) and 4.4% (1,268 genes) was significantly affected in BMM infected with the two *Mtb *strains at 6 and 24 hpi respectively (Figure 1B). In addition, expression of a higher number of shared host genes was significantly modulated by both strains at 6 hours (1,951 genes), compared to 24 hours of infection (831 genes). At 6 hpi, the number of differentially expressed host genes exclusively affected during infection by CDC1551 was about 3.5 fold higher (865 genes) than by HN878 infection (238 genes). However, while the total number of differentially expressed BMM genes following infection by HN878 was comparable between 6 and 24 hours (238 and 280 genes), a larger number of genes was induced at 24 hours by HN878, compared to CDC1551 infection (280 vs. 157 genes). The fold change in BMM gene expression between CDC1551 and HN878 infection at 6 hours ranged from +192.83 to -24.32 and from +157.62 to -21.95, respectively (Figure 1C and 1D). At 24 hpi, the expression levels were from +167.72 to -55.93 and from +172.62 to -40.62 in CDC1551 or HN878 infected BMM, respectively (Figure 1E and 1F). This robust modulation in gene expression by both *Mtb *strains indicated an early dynamic reprogramming of host cellular machinery upon infection of BMM by Mtb. Importantly, among the ten most highly up-regulated BMM genes, 6 and 7 genes were commonly expressed during infection by CDC1551 and HN878 at 6 and 24 hpi, respectively. Similarly, 7 and 6 genes out of the 10 most highly down-regulated host genes were common to infection with both CDC1551 and HN878 at 6 and 24 hpi, respectively (Additional file 1). This suggested that differential expression of the majority of macrophage genes was in response to the common determinants shared by both *Mtb *strains and only a subset of host genes were differentially expressed depending on the specific phenotype of the infecting bacilli. Overall, the global transcriptional analysis of the *Mtb*-infected BMM revealed that infection by CDC1551 induced a stronger macrophage response at 6 hpi, compared to HN878 which induced a lower but more protracted response that lasted at least 24 hpi.

### Biologic ontology analysis of differentially expressed macrophage genes during infection by *Mtb*

To gain insight into the cellular and molecular functions of the *Mtb*-induced host genes, differentially expressed macrophage genes from the microarray analysis were subjected to functional categorization using Ingenuity Pathway Analysis software (IPA; version 7.5) (Table 1) [18]. At 6 hpi, infection by CDC1551 of BMM modulated the expression of a higher number of host genes involved in the cell inflammatory response, cellular growth and proliferation, cell-to-cell signaling and interaction, cell death, gene expression, cell cycle, DNA replication and repair, compared to infection by HN878. However, by 24 hpi, HN878 modulated the expression of similar or even slightly higher numbers of host genes in the above mentioned functional categories (Table 1). Interestingly, similar numbers of host genes involved in antigen presentation were differentially expressed at 6 and 24 hpi of BMM by both HN878 and CDC1551. In addition, expression of a significantly higher number of host genes involved in lipid metabolism and small molecule biochemistry, including uptake of long chain fatty acids, synthesis of lipids, fatty acids and prostaglandins as well as cholesterol metabolism, was induced by HN878, compared to CDC1551 infection of BMM at 24 hours. The relative transcript levels of selected differentially expressed host genes, during infection of BMM by both *Mtb *strains and the respective functional category, can be found in Additional file 2.

**Table 1:** Ontology analysis of the differentially expressed macrophage genes by *Mtb *infection

Biological function	Number of differentially expressed genes			
	6 hours		24 hours	
	CDC1551	HN878	CDC1551	HN878
Inflammatory response	391	343	221	232
Cellular growth and proliferation	650	549	325	371
Cell-to-cell signaling and interaction	334	305	217	224
Antigen presentation	147	150	115	117
Cell death	512	455	243	279
Cell cycle	333	279	157	186
DNA replication, recombination and repair	212	141	54	69
Gene expression	507	399	161	178
Small molecule biochemistry	257	221	112	173
Lipid metabolism	211	188	112	150

Total host RNA was isolated from the BMM infected with *Mtb *CDC1551 or HN878 at 6 and 24 hpi and analyzed by microarray. The list of differentially expressed genes (at least 2 fold changes in expression levels and *P *≤ 0.05) was uploaded into IPA software for functional analysis. The number of significantly differentially expressed host genes upon *Mtb *infection at 6 and 24 hpi and their respective biological function are shown.

### Distinct gene networks modulated by CDC1551 and HN878 infection of macrophages

**Figure 2:**
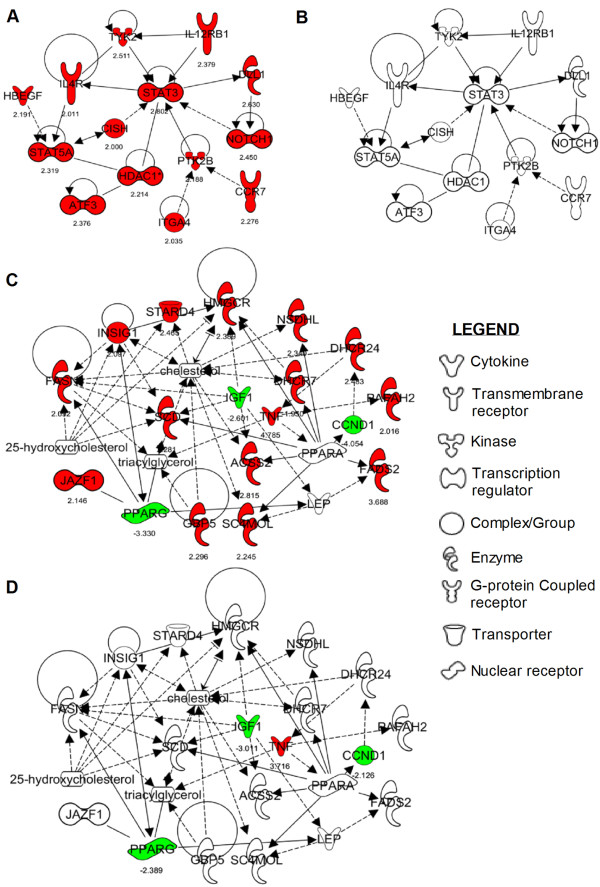
**Strain-specific host gene networks activated in *Mtb*-infected BMM**. Significantly differentially expressed host genes upon infection by CDC1551 or HN878 of BMM at 6 and 24 hpi were used to construct strain-specific networks by IPA. (A) Early immune activation network (EIAN); genes in this network were significantly up-regulated only in CDC1551 infected BMM at 6 hours. (B) Insignificant expression of EIAN genes during HN878 infection of BMM at 6 hours. (C and D) A subset of lipid metabolism pathway genes significantly activated only in HN878 infected BMM at 24 hours. (D) The majority of genes in the lipid metabolism pathway were not significantly expressed during CDC1551 infection of BMM at 24 hours. Up-regulated genes are marked red and down-regulated genes are marked green with their absolute level of expression under respective gene symbols (also described in Additional file 3). Solid lines indicate direct interaction and broken lines represent indirect interaction between the genes in the network.

Since the comparative transcriptome and gene ontology analysis of BMM infected with CDC1551 or HN878 suggested that at 6 hpi, CDC1551 modulated the expression of a higher number of genes involved in early immune activation pathways, and at 24 hpi, HN878 altered the expression of higher number of genes involved in host cell lipid metabolism, we focused on *Mtb *strain-specific networks within these two pathways. From the list of differentially expressed macrophage genes (at least 2-fold change in expression and *P *≤ 0.05), we identified using IPA, two networks that were specifically modulated in response to infection by one of the two *Mtb *strains. Expression of all the genes in the early immune activation network (EIAN) was up-regulated preferentially in CDC1551 infected BMM as early as 6 hours (Figure 2A and 2B), while expression of the EIAN genes was not significant in the HN878 infected BMM at 6 hours (Figure 2B and Additional file 3). The EIAN is a branch-network of the main host immune response pathway that is commonly activated upon infection by both *Mtb *strains. The genes in EIAN encode immune regulatory and cell signaling molecules, such as STAT3, STAT5A, ATF3, HDAC1, PTK2B, CISH and TYK2, as well as cytokine and chemokine receptors, including IL4R, IL12RB1, NOTCH1, ITGA4 and CCR7. The second network, comprised of a subset of genes involved in host lipid metabolism, was up-regulated specifically during HN878 infection of BMM at 24 hours (Figure 2C and 2D). The majority of the genes in this network are involved in cholesterol metabolism (*Fasn*, *Insig1*, *Stard4*, *Hmgcr*, *Nsdhl*, *Dhcr24*, *Dhcr7*, *Acss2*, *Sc4mol*, *Gbp5 *and *Scd*). However, subsets of these genes are also involved in 25-hydroxycholesterol metabolism (*Fasn*, *Insig1*, *Stard4*, and *Hmgcr*) and triacylglycerol metabolism (*Fasn *and *Gbp5*). Importantly, though four genes of this network, (*Pparg*, *Igf1*, *Ccnd1 *and *Tnf*) that are part of the general lipid metabolism pathway, were expressed similarly in both CDC1551 or HN878 infected BMM at 24 hours, the absence of significant regulation of other key genes in this lipid metabolism network in CDC1551 infected BMM was striking.

### Validation of differentially expressed host genes by qRT-PCR

**Figure 3:**
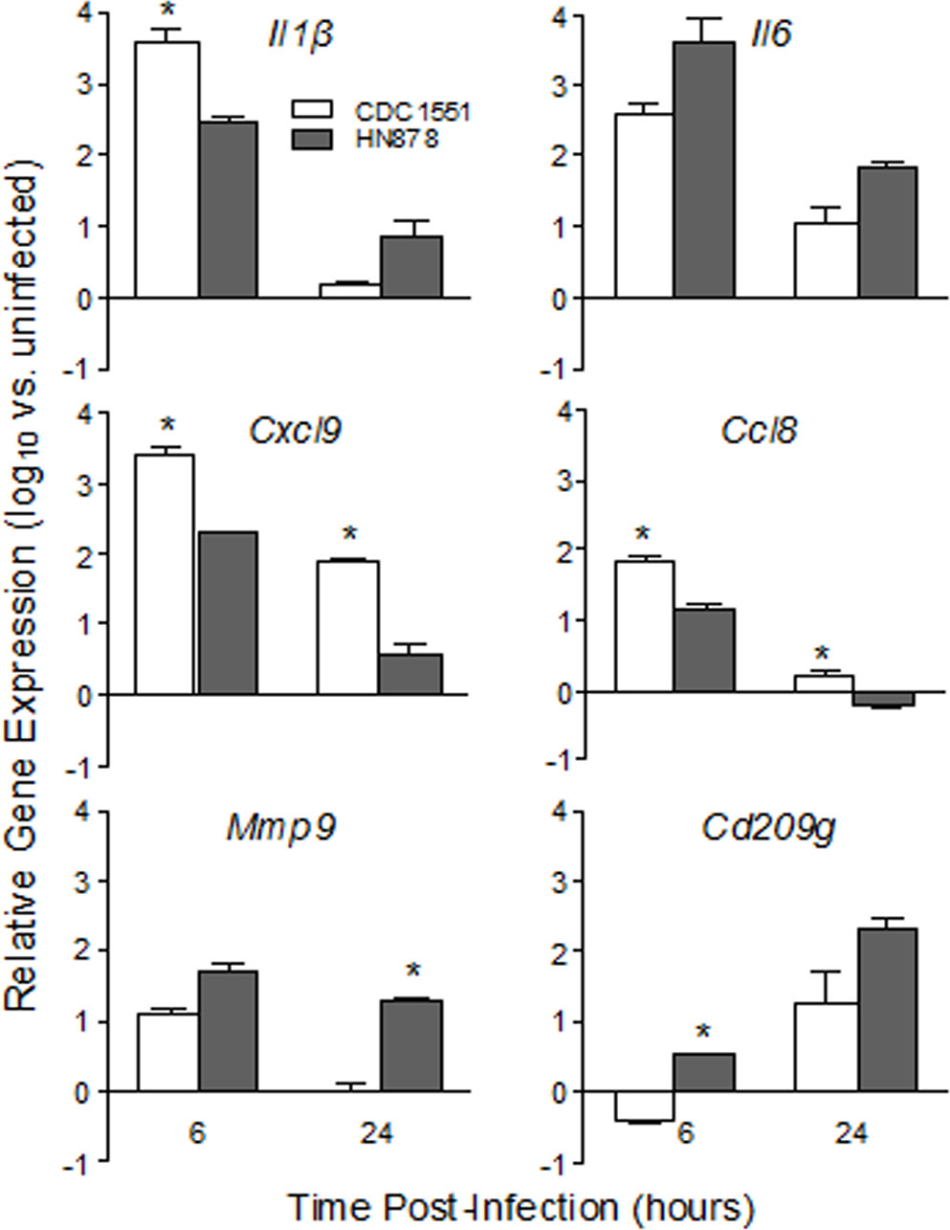
**Expression of cytokine, chemokine and inflammatory genes during *Mtb *infection of BMM**. Total RNA from BMM infected with CDC1551 or HN878 at 6 and 24 hpi was used to quantify the transcript levels of cytokines (*Il1β *and *Il6*), chemokines (*Cxcl9 *and *Ccl8*), and inflammatory molecules (*Mmp9 *and *Cd209g*) by qRT-PCR. The threshold cycle (C_t_) of each test gene was normalized against that of the house-keeping gene, *Gapdh *and the relative fold change in gene expression was calculated by comparing *Mtb*-infected and uninfected samples. The data presented are average ± standard deviation of values obtained from three independent infections per experimental group assayed at least in duplicate. * *P *≤ 0.05.

To validate the microarray gene expression profile of BMM during infection by *Mtb *, we performed qRT-PCR for selected genes encoding mediators of host immunity, including pro-inflammatory cytokines (IL-1β and IL-6), chemokines (CXCL9 and CCL8), cell adhesion and tissue remodeling (MMP9 and CD209F) and host lipid metabolism genes (Figure 3 and Table 2). Expression of *Cxcl9 *and *Ccl8 *was significantly up-regulated in CDC1551-infected cells at both 6 and 24 hpi, compared to infection by HN878, while *Il1β *was more highly up-regulated at 6 hours than at 24 hours (Figure 3). In contrast, while *Il6 *and *Mmp9 *were significantly highly expressed in HN878-infected cells at both time points, the level of expression of *Cd209g* was similar at 6 hours and higher in HN878-infected BMM at 24 hours of infection. Similar to the microarray data, the relative expression ratio between HN878 and CDC1551, of several host genes involved in lipid metabolism, measured by qRT-PCR was higher in HN878 infected BMM at 24 hpi (Table 2). Taken together, the results from qRT-PCR analysis were consistent with the microarray data and confirm our conclusion that CDC1551 infection of BMM selectively triggers EIAN at 6 hours, while infection by HN878 selectively up-regulates a host cell lipid metabolism network at 24 hpi.

**Table 2:** Transcript levels of macrophage genes involved in lipid metabolism altered by *Mtb *infection

Gene	Annotation	Expression Ratio (HN878/CDC1551)		Function
		*Micro* ***Array***^**§**^	***qRT-PCR***^**€**^	
*Scd1*	Steroyl-CoA desaturase 1	1.85	12.13*	Fatty acid synthesis
*Fads2*	Fatty acid desaturase 2	2.95	8.51*	Lipid metabolism
*Gpd2*	Glycerol-3-phosphate dehydrogenase	1.35	3.61*	Glycerolphospholipid metabolism
*Adora2b*	Adenosine A2b receptor	1.63	3.59*	Lipid synthesis
*Fdft1*	Farnesyl diphosphate fanesyltransferase 1	1.53	2.77*	Cholesterol synthesis
*Gbgt1*	Globoside alpha-1,3-N-acetogalatosaminyltransferase 1	1.64	2.75*	Glycolipid metabolism
*Acsl1*	Acyl-CoA synthetase long chain family member 1	1.71	2.69*	Fatty acid synthesis
*Acss2*	Acyl-CoA synthetase short chain family member 1	1.53	2.58*	Lipid synthesis
*Hsd17b7*	Hydroxysteroid 17-beta dehydrogenase 7	1.39	2.58	prostaglandin metabolism
*Lss1*	Lanosterol synthase 1	1.34	2.51*	Fatty acid metabolism
*Dhcr24*	24-dehydrocholestrol reductase	2.30	2.36*	Cholesterol synthesis
*Ppap2b*	Phosphatidic acid phosphatase type 2B	1.35	2.26*	Lipid synthesis
*Acat2*	Acyl-CoA thioesterase 7	1.43	1.82*	Fatty acid metabolism
*Scd2*	Steroyl-CoA desaturase 2	1.63	1.80	Fatty acid synthesis
*Mvd*	Mevalonate decarboxylase	1.42	1.72*	Cholesterol synthesis
*Fdps*	Farnesyl diphosphate synthase	1.30	1.64	Sterol synthesis
*Ptges*	Prostaglandin E synthase	1.92	1.60*	Prostaglandin metabolism
*Cyp51*	Cytochrome P450, family 51, subfamily A, polypeptide 1	1.50	1.35*	Sterol synthesis
*Idi1*	Isopentenyl diphosphate delta isomerase 1	1.69	ND	Cholesterol synthesis

^§ ^values are averages compiled from at least 3 independent experiments with each *Mtb *strain.

^€ ^values are averages compiled from at least 3 independent experiments and differentially expressed macrophage genes between CDC1551 and HN878 infection that are statistically significant by both microarray and qRT-PCR are marked with an asterisk (*).

Significantly differentially expressed macrophage genes involved in lipid metabolism at 24 hpi by *Mtb *CDC1551 or HN878. Levels of gene expression were derived from microarray and qRT-PCR experiments. Relative gene expression ratio for microarray and qRT-PCR was determined by dividing the expression values from HN878-infected BMM by that of CDC1551-infected BMM. ND- transcript not detected.

### Nitrite (NO_2_^-^) production by *Mtb *infected macrophages

**Figure 4:**
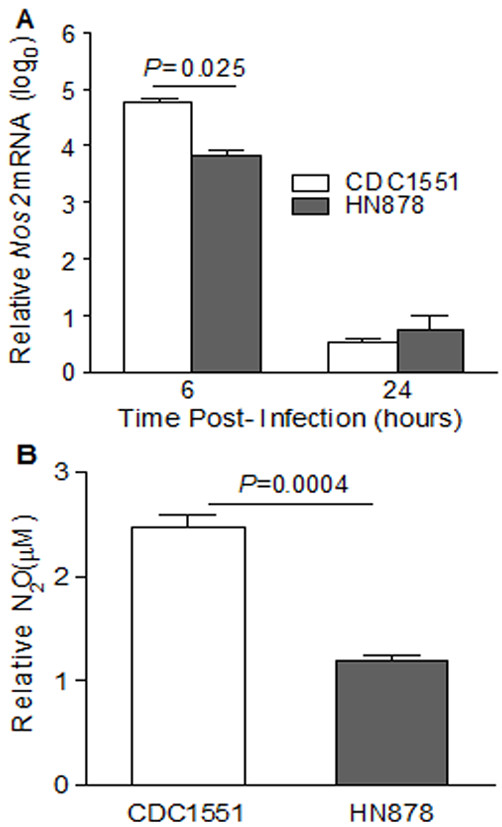
**Transcript levels of *Nos2 *and production of nitric oxide by *Mtb*-infected BMM**. (A) Total host RNA from CDC1551 or HN878- infected BMM at 6 and 24 hpi was used to quantify the levels of *Nos2 *mRNA levels. The expression levels were normalized to *Gapdh *levels from the same sample and relative fold change was calculated by comparing uninfected with 6 and 24 hpi samples. (B) Production of nitric oxide measured as dissolved nitrite (NO_2_^-^) using Griess reagent as described in methods. The culture supernatants from BMM infected with CDC1551 or HN878 were collected 24 hpi and the amount of NO_2_^- ^was measured in triplicate samples per infection. Relative NO_2_^- ^concentrations were calculated by extrapolation of values from test samples on standard curves generated using sodium nitrite. Data represent mean ± standard error from two independent experiments performed in triplicate for each experimental group.

We observed that the level of expression of nitric oxide synthase (*Nos2*), a marker of macrophage activation, was significantly higher in CDC1551-infected BMM, compared to infection by HN878 (Figure 4A and Additional file 1). Therefore, we measured the NO_2_^- ^accumulation in the culture supernatants of BMM infected with both *Mtb *strains. Consistent with the microarray results, the concentration of NO_2_^- ^at 24 hpi was more than two-fold higher in response to infection by CDC1551, compared to HN878 (Figure 4B). However, there was no change in the nitrite production upon infection by HN878 and CDC1551 at 6 hpi. These results suggest that, compared to HN878, CDC1551-infected BMM were more effectively activated. This observation also suggests that the more activated BMM generate higher levels of the anti-microbial mediators and thus impose a more hostile intracellular environment on the bacteria.

### Transcription of mycobacterial stress response genes during infection of macrophages

**Figure 5:**
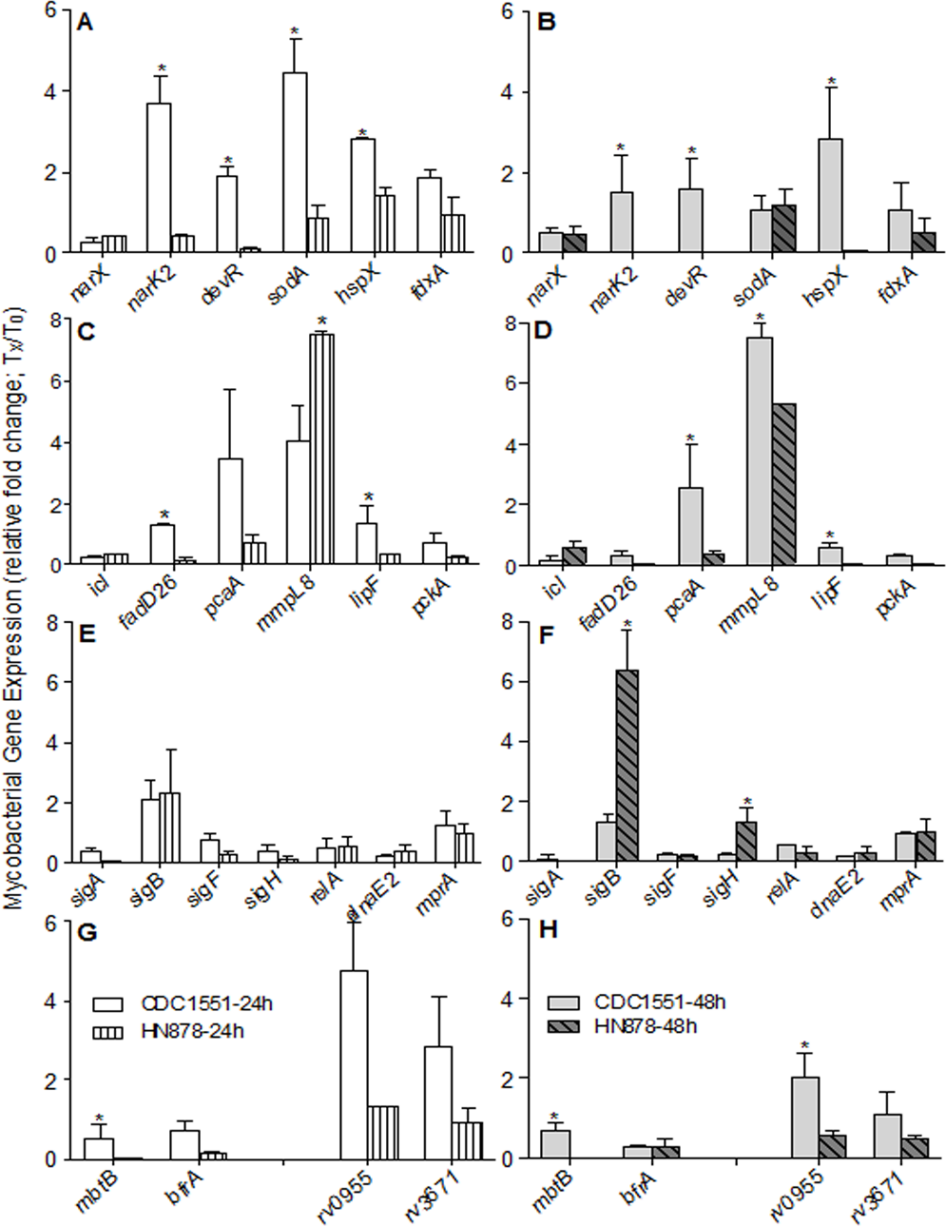
**Differential expression of *Mtb *genes during BMM infection**. Total *Mtb *RNA was isolated from CDC1551 or HN878-infected BMM at 24 and 48 hpi and the level of expression of *Mtb *genes involved in pathways including hypoxia, general stress response and fatty acid/lipid metabolism was determined by qRT-PCR. The amount of transcripts for each *Mtb *gene was normalized to 16S rRNA levels and relative fold change in gene expression was calculated by comparing the expression level at 24 and 48 hpi with T0 levels. (A and B) Intracellular mRNA levels of *Mtb *genes involved in mycobacterial survival during hypoxia expressed by CDC1551 or HN878 at 24 (A) and 48 (B) hpi. (C and D) Transcript levels of *Mtb *genes involved in fatty acid/lipid metabolism expressed by CDC1551 or HN878 at 24 (C) and 48 (D) hpi of BMM. (E and F) mRNA levels of *Mtb *genes involved in general stress response expressed by intracellular CDC1551 or HN878 at 24 (E) and 48 (F) hpi. (G and H) Intracellular mRNA levels of *Mtb *genes involved in iron metabolism and peptidases involved in acid shock response expressed by CDC1551 or HN878 at 24 (G) and 48 (H) hpi. The results are means ± standard deviation from three independent samples per experimental group per time point repeated at least twice. * *P *≤ 0.05.

We next analyzed the intracellular bacterial transcriptional response during CDC1551 or HN878 infection of BMM (Figure 5). Previous studies have described the host and bacterial transcriptional profiles of diverse lineages of clinical *Mtb *isolates in response to macrophage infection [19-21]. From those reports, we selected a subset of *Mtb *genes and determined their level of expression by qRT-PCR. These *Mtb *genes are known to be modulated in response to intracellular stress, such as hypoxia/anaerobiosis (*narX, narK2, devR *(also known as *dosR*)*, sodA, hspX *and *fdxA*), acid shock (*Rv0955 and Rv3671*) and general stress (*sigA, sigB, sigF, sigH, relA, dnaE2 *and *mprA*) as well as cell wall biosynthesis/lipid metabolism (*icl, fadD26, pcaA, mmpL8, lipF *and *pckA*), and iron metabolism/storage genes (*mbtB *and *bfrA*) [21-24]. In both *Mtb *strains, the intracellular transcription of the majority of the tested genes from all categories was up-regulated at 24 hours and either stayed at similar levels or was slightly reduced at 48 hpi of BMM. However, intracellular expression of *mmpL8 *in CDC1551 and *sigB *and *sigH *in HN878 were gradually up-regulated from 24 to 48 hpi. In addition, the transcript levels of four (*narK2*, *devR*, *sodA *and *hspX*) and three (*narK2*, *devR *and *hspX*) out of six tested *Mtb *genes, induced by anaerobic stress were significantly higher in intracellular CDC1551 than HN878 at both 24 and 48 hpi (Figure 5). In contrast, the transcript levels at 24 and 48 hpi, of *narX*, *fdxA *and *sodA *at 48 hpi were similar between CDC1551 and HN878 (Figure 5A and 5B). The transcripts of all tested cell wall biosynthesis/lipid metabolism genes, except for *icl *and *mmpL8*, were also higher in intracellular CDC1551 than in HN878 (Figure 5C and 5D). While the levels of *icl *transcripts were similar, the kinetics of expression of *mmpL8 *was different for the two *Mtb *strains. In addition, the expression of *Mtb *genes encoding alternative sigma factors (*sigB*, *sigF *and *sigH*) and general stress response (*relA*, *dnaE2 *and *mprA*) were similarly regulated at 24 hpi in both *Mtb *strains, while higher *sigB *and *sigH *transcripts were expressed by intracellular HN878 at 48 hpi, compared to CDC1551 (Figure 5E and 5F). Finally, the intracellular expression of *Mtb *genes involved in iron uptake/storage (*mbtB *and *bfrA*) and acid shock response (*Rv0955 *and *Rv3671*) were also significantly up-regulated in CDC1551, compared to HN878 at both 24 and 48 hpi (Figure 5G and 5H). There was no significant difference in the basal level expression of most of the tested genes between CDC1551 and HN878 (Additional file 4). However, the basal levels of *narX*, *narK2*, *bfrA*, *fdxA*, *sigB*, *sodA*, *Rv3671 *and *Rv0955 *transcripts in CDC1551, and *devR *(*dosR*) levels in HN878 was significantly higher compared to their counter parts. Thus, in the more extensively activated BMM (those infected with CDC1551), the intracellular stress on the bacilli was greater, resulting in significantly higher expression of several *Mtb *stress response genes.

## Discussion

In this study, we explored the transcriptional response of BMM to infection by *Mtb *CDC1551 or HN878 to elucidate the molecular basis for their differential response in the mouse and rabbit models of pulmonary TB [6,12,25]. The global transcriptome of *Mtb*-infected BMM showed distinct patterns of gene expression. Compared to HN878, a more robust and rapid change in the host transcriptional response was elicited upon infection by CDC1551 as early as 6 hours; thereafter the number of differentially expressed BMM genes induced by CDC1551 infection was reduced by 24 hpi. In contrast, the transcriptional response of BMM to infection with HN878 was sub-optimal at the early time-point (6 hours) and showed a more protracted profile at 24 hpi.

The differential activation of BMM by CDC1551 infection was associated with the specific up-regulation of genes that constitute EIAN which is part of the global host immune response. The majority of EIAN genes, such as *Stat3*, *Stat5a*, *Atf3*, *Hdac1*, *Ptk2b*, *Cish *and *Tyk2 *are transcriptional regulators that control immune cell functions including cell maturation, proliferation, survival, migration and antimicrobial defense [26-32]. For example, STAT3 and STAT5A, activated by different types of cytokines in a cell-specific manner, affect the differentiation, growth and apoptosis of host cells by controlling the expression of key genes in respective cellular pathways; STAT3 activation down-regulates IGFBP5, a growth factor binding protein that interacts with IGF1 and promotes apoptosis of T cells. In addition, activation of STAT3 enhances proliferation of various immune cells through a gp130-mediated pathway [26,33,34]. Mice that harbor a conditional disruption of *Stat3 *in macrophages and neutrophils were shown to have elevated levels of inflammatory cytokines in the serum and were more vulnerable to the effects of lipopolysaccharide (LPS)-induced shock [26]. Tyrosine kinase-2 (TYK2), one of the interacting partners of STAT3, also plays a crucial role in macrophage activation by various stimulants such as cytokines and LPS [27,28,35]. Accordingly, *Tyk2 *knockout (KO) mice were defective for IFN-γ signaling and for protective T-cell responses against lymphocytic choriomeningitis virus infection [28]. Moreover, macrophages of the *Tyk2*-KO mice neither expressed *Nos2 *nor produced NO in response to LPS stimulation [28,35]. In addition, these mutant mice displayed reduced production of IL-12 and STAT3 activation [28]. Furthermore, a proline-rich tyrosine kinase (PTK2B alias PYK2) in the EIAN, up-regulated during CDC1551 infection of BMM, has been shown to mediate the NADPH oxidase-dependent generation of reactive oxygen intermediates (ROI) and *Pyk2*-KO mice showed defective ROI production and associated host pro-inflammatory molecules, including TNF-α and MCP-1 [36]. Similarly, NO production by highly activated macrophages has been shown to mediate mycobacterial killing by direct toxicity and indirect interference with *Mtb *virulence determinants [37,38].

We also observed increased level of expression of DC-SIGN (dendritic cell-specific ICAM-3 grabbing non-integrin; also designated as CD209) upon infection of BMM with HN878 compared to CDC1551. CD209 has been shown to play a key role in the dissemination of HIV-1 by dendritic cells (DCs) [39]. In addition, DC-SIGN mediates adherence of *Mtb *to human macrophages and DCs and interferes with the cellular inflammatory response to infection by *Mtb *[40,41]. DC-SIGN binding to ManLAM, a virulence factor secreted by *Mtb-*infected DCs, prevents DC maturation by compromising Toll-like receptor (TLR)-mediated signaling [40]. These observations, combined with the lower NO produced by HN878 infected BMM support our conclusion that HN878 is less efficient at inducing macrophage activation and bactericidal responses. Our present findings are also supported by a recent report from Portevin *et.al*, that showed differential cytokine and inflammatory responses of human macrophages upon infection by ancient versus modern lineage clinical *Mtb *strains; variable early inflammatory responses induced by these *Mtb *strains were suggested to contribute to the difference in their pathogenicity [42]. Taken together, the early macrophage activation in response to infection by CDC1551 may explain the observation made in infected rabbits where CDC1551 reached a relatively low maximal bacillary load (5.5-6 log_10_) in the lungs and the CFU declined gradually to complete clearance at 3-4 months. In contrast, infection by HN878 of rabbits led to extensive *Mtb *growth reaching a higher and sustained bacillary load (7-9 log_10_) in the lungs. Thus, early and robust macrophage activation appears to be essential for effective control of infection by *Mtb *and for prevention of progression to active granulomatous disease [6,12].

In our study, we noted that infection of BMM with HN878 up-regulated the expression of a larger number of host lipid metabolism genes, including *Acsl1*, *Dhcr24*, *Acat2*, *Scd1 *and *Fads2*. Recent studies from others and our laboratory have shown that infection by *Mtb *induces lipid droplet formation in human macrophages [43], and that elevated host lipid metabolism correlates with caseation in human TB granulomas [7,17]. Moreover, only pathogenic *Mtb *could induce the differentiation of phagocytes into foamy macrophages (FMs) [43]. The association between altered lipid metabolism and pathogenesis has also been reported in infection by other microbes, such as *Toxoplasma gondii *[44]. In mouse stromal vesicular cells that displayed foam cell morphology, expression of *Acsl1*, coding for an acyl-CoA synthase, which mediates fatty acid homeostasis and cell apoptosis, was up-regulated [45]. Similarly, *Dhcr24 *encoding dihydroxy cholesterol reductase has been shown to facilitate replication of hepatitis C virus in mouse hepatocytes and *Acat*, coding for acyl-CoA thioesterase, involved in cholesterol biosynthesis, has been reported to be required for the replication of novovirus in HG23 cells [46,47]. However, a role for DHCR24 and ACAT in bacterial infections has not been reported before. It has been shown that cholesterol is essential for the uptake of *Mtb *by primary human and mouse macrophages [48] and catabolism of cholesterol has been reported to be essential for the persistence of *Mtb *during chronic infection of mice [49]. In addition, elevated levels of host cholesterol and/or fatty acids have been shown to be essential for the persistence of *Mtb *in granulomas presumably by preventing phagosome maturation [50]. High levels of prostaglandin E2 (PGE2) have a significant immunosuppressive effect, such as inhibition of lymphocyte proliferation and production of Th1 type cytokines as well as interference with macrophage activation [51,52]. Strong PGE2 immunostaining was seen in the FMs of H37Rv-infected mouse lungs [53]. Consistent with these results, the up-regulation of *Ptgs2 *expression in HN878-infected BMM could lead to suppression of macrophage-mediated innate responses and enhance FM formation during chronic HN878 infection *in vivo*. Taken together, these studies may help explain our findings in the rabbit model of pulmonary TB, where infection by CDC1551 was efficiently controlled while infection by HN878 progressed to chronic granulomatous disease [6,11,12,18].

Both the host and pathogen response during infection contribute to the balance that determines the outcome in TB [2,54,55]. The level of intracellular expression of *Mtb *stress-response genes upon infection has been shown to reflect the extent of immune pressure exerted by the host immune response. Whereas the early and highly activated BMM induced by CDC1551 infection triggered a rapid and robust *Mtb *stress response, the sub-optimal activation of BMM upon infection by HN878 resulted in diminished environmental pressure on the bacteria [19,21]. We observed higher expression levels of hypoxia/anaerobiosis-induced genes (*narK2, devR, hspX*, *sodA*, *fdxA *and *icl*) during intracellular infection with CDC1551 compared to HN878. Several *in vitro *and *in vivo *studies have examined the role of these genes in *Mtb *pathogenesis and persistence [56-58]. It is well documented that DosR, the master regulator of "DosR regulon" regulates the expression of several genes, including *narX*, *narK2*, *hspX*, *fdxA*, *pcaA *and *devR *that are induced when *Mtb *encounters hypoxic conditions [59,60]. Earlier reports have shown that the level of expression of many DosR regulon genes, including *dosR *itself, is up-regulated during the bacterial adaptation to dormancy and/or microaerophilic growth and that mutations in these genes resulted in increased bacillary growth both *in vitro *and in the lungs and spleens of infected mice, highlighting the essentiality of these genes for intracellular mycobacterial adaptation in the host [21,61-64]. However, growth of *dosR *mutant in the lungs of *Mtb*-infected experimental animals is highly variable, ranging from attenuated (in mice and guinea pigs) to no change in growth (in rabbits and mouse "hollow-fiber" model), compared to wild type controls [65]. Similarly, *pcaA*, encoding cyclopropane synthase is reported to be essential and sufficient for early activation of mouse BMM and growth of the *pcaA *mutant was exacerbated in the infected mouse lungs [66,67]. Moreover, *Mtb *genes *Rv3671c *and *Rv0955 *were shown to protect the intracellular bacilli from acid shock and oxidative stress during infection of activated macrophages and a mutation in these genes renders the bacilli severely attenuated for growth in infected mouse lungs [68,69]. Thus, up-regulation of the DosR regulon genes in CDC1551 clearly supports our conclusion that, in contrast with HN878, this strain is exposed to enhanced intracellular pressure, exerted by the highly activated BMM.

## Conclusions

Our data on the modulation of host and bacterial gene expression upon CDC1551 versus HN878 infection of BMM suggest that even relatively subtle differences in the expression of genes that regulate immune cell activation, induced very early in infection by CDC1551 was significant enough to impact the intracellular stress of the bacilli. Our results support the link between specific host-derived, intracellular signals and the modulation of bacterial gene expression [20,70-72]. We propose that an early induction of the immune activation network and stronger BMM activation by CDC1551 infection can ultimately result in efficient control of the bacilli by host cells. In contrast, sub-optimal BMM activation, associated with elevated host lipid metabolism that contributes to FM formation, enables better intracellular survival of the infecting HN878. Future studies will dissect in more detail the association between *Mtb *virulence and host lipid metabolism.

## Methods

### Bacterial culture

*Mycobacterium tuberculosis *CDC1551 and HN878 were grown in Middlebrook 7H9 medium supplemented with Middlebrook 10% oleic acid albumin dextrose catalase (OADC) enrichment (Difco BD, Franklin Lakes, NJ), 0.5% glycerol and 0.05% Tween 80 (Sigma-Aldrich, St. Louis, MO) at 37°C to logarithmic phase (OD_540 _= 0.6-0.7). Bacterial stocks were stored at -80°C until use. Bacillary clumping was prevented by probe sonication for 10 sec before infection.

### Macrophage infection and bacterial load determination

BMM were generated from the marrow of femur bones of 8 week old female B6D2F1 mice (Jackson Laboratories, Bar Harbor, ME) as described earlier [73]. Briefly, BMM were differentiated in Dulbecco's Modified Eagle Medium (Gibco, Grand Island, NY) supplemented with 10% Fetal Bovine Serum, sodium pyruvate, 1% L-glutamine, 20% L929 cell-conditioned medium and 1% penicillin/streptomycin and cultured in a 5% CO_2 _incubator for 7 days. Differentiated BMM were infected with CDC1551 or HN878 at a multiplicity of infection (MOI) of 5:1 (bacteria: BMM). After 3 hours incubation at 37°C, extracellular bacteria were removed by washing three times with sterile phosphate buffered saline (PBS). Uninfected and *Mtb*-infected cell cultures were incubated up to 48 hours and supernatants were harvested at 24 and 48 hpi for further analysis. For bacterial load enumeration, infected cells were lysed by sonication and serial dilutions in sterile PBS-Tween 80 were spread on Middlebrook 7H10 agar plate (Difco BD, Franklin Lakes, NJ) and number of bacterial colonies (CFU) were counted after 3-4 weeks of incubation at 37°C [18].

### Measurement of nitrite (NO_2_^-^) production

Nitrite (NO_2_^-^) levels were measured to determine the amount of dissolved NO produced by cells in the supernatants of uninfected and *Mtb*-infected BMM using Griess reagent as described previously [74]. Briefly, 100 μl of supernatant in a 96-well plate was mixed with an equal volume of Griess reagent (Sigma-Aldrich, St. Louis, MO) and incubated for 10 minutes at room temperature in the dark. The color developed was measured at a wavelength of 540 nm in a spectrophotometer (Versa max; Molecular Devices, Sunnyvale, CA). The concentration of NO_2_^- ^in the test samples was derived from the standard curves obtained with known concentration of sodium nitrite, run in parallel with the test samples. Each experiment was repeated in triplicate using samples from three independent experiments.

### Total RNA isolation and microarray analysis of *Mtb *infected macrophages

Total BMM RNA was obtained from *Mtb*-infected cells at 6 and 24 hpi using the Trizol method as mentioned previously [18]. Briefly, BMM were lysed with Trizol reagent (Invitrogen, Carlsbad, CA) and total RNA was isolated with RNeasy mini kit according to the manufacturer's instruction (Qiagen, Valencia, CA). RNA from uninfected BMM was included as control. Total *Mtb *RNA was isolated using a modified differential lysis method. Briefly, infected cells were lysed in sterile 0.1% Triton X-100, followed by centrifugation at 13,000 rpm for 10 min at 15°C to pellet the bacteria. The bacterial pellet was re-suspended in Trizol and subjected to bead beating with Ribo-lyser (MP Biosciences, Solon, OH) for 2 min as 30 sec pulses with 1 min ice-incubation in between the pulses. The host and bacterial RNA were subjected to DNaseI digestion before final purification through RNeasy mini kit (Qiagen, Valencia, CA). For the microarray experiments, total host RNA (300 ng) was reverse transcribed and labeled with Cy3 and Cy5 using the Fluorescent liner amplification kit according to manufacturer's instructions (Affymetrix, Santa Redwood city, CA). Synthesized cDNA was hybridized to the Affymetrix mouse GeneChip Gene ST 1.0 as described previously [18]. Three independent RNA samples were obtained per experimental group and used for microarray experiments.

### Microarray data analysis of macrophage gene expression

Probe-level intensity measurement from the microarray chip composed of 28,853 annotated genes was normalized using the Robust Multi-array Average (RMA) method and summarized using the Partek Genomics Suite platform (Partek Inc., St. Louis, MO). The raw data from microarray experiments (CEL files) contains the expression level (intensity) of probe sets. The signal intensity from each probe set was subtracted from the background values. The gene expression ratio (infected vs. uninfected) was calculated as median-centered values (from 3 independent experiments) and denoted as log2 values. However, when the expression ratio was converted to fold change, the log2 values were transformed to normal (log10) values. The annotated and differentially expressed genes were identified based on changes in average expression levels with a significance of *P *≤ 0.05. The genes differentially expressed in response to infection by *Mtb *were identified by calculating the ratio of gene expression between infected and uninfected, control cells. Genes with *P *≤ 0.05 and at least two fold changes in the level of expression were regarded as significantly differentially expressed. Microarray data of this study are available in the Gene Expression Omnibus (GEO) repository (accession number GSE31734). List of significantly differentially expressed host genes at 6 and 24 hpi was uploaded to Ingenuity Pathway Analysis (IPA; version 7.5) software (Ingenuity Systems, Redwood city, CA) for gene ontology analysis, functional classification and network derivation of differentially expressed genes.

### Quantitative real time RT-PCR (qRT-PCR) analysis

Differential expression of the selected host and bacterial gene expression was determined by qRT-PCR using SYBR Green-ER two-step qRT-PCR kit (Invitrogen, Carlsbad, CA). The cDNA was amplified with gene specific primers. The nucleotide sequences of primers specific to mouse genes were obtained from http://pga.mgh.harvard.edu/primerbank/. The DNA sequences of the mycobacterial primers used for qRT-PCR have been reported earlier [21]. The qRT-PCR was performed in the MxPro4000 Multiplex quantitative PCR System (Stratagene, Santa Clara, CA). The threshold cycle (Ct value) for each amplified target gene was calculated using MxPro4000 software. Uniform baseline fluorescence was set for all the genes in each experiment and across different experiments. The transcripts of glyceraldehyde phosphate dehydrogenase (*Gapdh*) for mouse genes and 16S rRNA for the *Mtb *genes were used to normalize the Ct values of the target genes. Fold change in gene expression was calculated using the formula 2^-ΔΔCt ^and represented as relative expression after normalization to uninfected group. The experiments were repeated three times with RNA samples from two to three wells per experimental group and time point.

### Statistical analysis

All values are presented as mean ± standard deviation or mean ± standard error of means of two to three independent experiments with multiple samples. Comparisons between experimental conditions were analyzed by the Student's *t*- test. Differences were considered statistically significant when *P *≤ 0.05.

## List of abbreviations

BMM: bone marrow-derived macrophage; TB: tuberculosis; *Mtb*: *Mycobacterium tuberculosis*; PGL: phenolic glycolipid; EIAN: early immune activation network; NO: nitric oxide; IPA: ingenuity pathway analysis; ROI: reactive oxygen intermediates; DC-dendritic cell; FM: foamy macrophage; TLR: Toll-like receptor; GEO: Gene Expression Omnibus; RMA: robust multi-array average; LPS: lipopolysaccharide; hpi: hours post-infection; KO: knock-out; Ct: threshold cycle; CFU: colony forming units; qRT-PCR: quantitative, real-time polymerase chain reaction; OADC: oleic acid albumin dextrose catalase.

## Competing interests

The authors declare that they have no competing interests.

## Authors' contributions

MSK, SS and GK conceived and designed the study; MSK and SS performed the experiments; MSK, SS and GK analyzed the data and wrote the manuscript. All authors read and approved the final manuscript.

## Supplementary Material

Additional file 1 **Top ten differentially expressed host genes by CDC1551 and HN878 infection at 6 and 24 hours**. The top 10 differentially expressed macrophage genes during infection by CDC1551 or HN878 at 6 and 24 hours were grouped according to their level of expression and significance (*P *value). The macrophage genes commonly up-regulated at 6 hpi between CDC1551 and HN878 are highlighted in red; commonly down-regulated at 6 hpi are marked in green; commonly up-regulated at 24 hpi are shown in purple color and commonly down-regulated at 24 hpi are denoted in blue. Click here for file

Additional file 2 **Relative transcript levels of key macrophage genes modulated during infection by *Mtb***. The macrophage genes differentially expressed upon infection by CDC1551 or HN878 at 6 and 24 hpi were derived from the microarray analysis and their expression ratio was calculated by dividing the mRNA level of CDC1551- infected with that of HN878-infected BMM. The genes are grouped based on their biological function and ranked according to their ratio within the functional category. Click here for file

Additional file 3 **Expression levels of macrophage EIAN and lipid metabolism genes during infection by *Mtb***. The differentially expressed host genes upon BMM infection with CDC1551 or HN878 at 6 and 24 hpi were identified by microarray analysis and subjected to functional pathway analysis using IPA. Two networks, an early immune activation network (EIAN) at 6 hpi and a sub-network of lipid metabolism pathway at 24 hpi, were differentially modulated in CDC1551 or HN878 infected BMM, respectively. The fold changes in the level of expression of genes that constitute those two networks are shown. Click here for file

Additional file 4 **Basal level expression of mycobacterial genes upon infection of BMDM**. Total RNA was isolated from CDC1551 or HN878 after 3 h infection of BMM (T0) and the level of expression of *Mtb *genes involved in hypoxia, general stress response and fatty acid/lipid metabolism pathways was determined by qRT-PCR. The amount of transcripts for each *Mtb *gene was normalized to 16S rRNA levels. The results are means ± standard deviation from three independent samples repeated at least twice. * *P *≤ 0.05. Click here for file

## References

[B1] Boshoff HI Barry CE Tuberculosis - metabolism and respiration in the absence of growth Nat Rev Microbiol 2005 3 1 70 80 10.1038/nrmicro1065 15608701

[B2] Cardona PJ Ivanyi J The secret trumps, impelling the pathogenicity of tubercle bacilli Enferm Infecc Microbiol Clin 2011 29 Suppl 1 14 9 21420562 10.1016/S0213-005X(11)70013-1

[B3] Karakousis PC Bishai WR Dorman SE Mycobacterium tuberculosis cell envelope lipids and the host immune response Cell Microbiol 2004 6 2 105 16 10.1046/j.1462-5822.2003.00351.x 14706097

[B4] Sturgill-Koszycki S Schlesinger PH Chakraborty P Haddix PL Collins HL Fok AK Allen RD Gluck SL Heuser J Russell DG Lack of acidification in Mycobacterium phagosomes produced by exclusion of the vesicular proton-ATPase Science 1994 263 5147 678 81 10.1126/science.8303277 8303277

[B5] Jayachandran R Sundaramurthy V Combaluzier B Mueller P Korf H Huygen K Miyazaki T Albrecht I Massner J Pieters J Survival of mycobacteria in macrophages is mediated by coronin 1-dependent activation of calcineurin Cell 2007 130 1 37 50 10.1016/j.cell.2007.04.043 17632055

[B6] Kaplan G Tsenova L Leong FJ, Dartois V, Dick T Pulmonary tuberculosis in the rabbit A color atlas of comparative pathology of pulmonary tuberculosis 2010 Boca Raton: CRC Press 107 129

[B7] Peyron P Vaubourgeix J Poquet Y Levillain F Botanch C Bardou F Daffe M Emile JF Marchou B Cardona PJ de Chastellier C Altare F Foamy macrophages from tuberculous patients' granulomas constitute a nutrient-rich reservoir for M. tuberculosis persistence PLoS Pathog 2008 4 11 e1000204 10.1371/journal.ppat.1000204 19002241 PMC2575403

[B8] Russell DG Cardona PJ Kim MJ Allain S Altare F Foamy macrophages and the progression of the human tuberculosis granuloma Nat Immunol 2009 10 9 943 8 10.1038/ni.1781 19692995 PMC2759071

[B9] Segovia-Juarez JL Ganguli S Kirschner D Identifying control mechanisms of granuloma formation during M. tuberculosis infection using an agent-based model J Theor Biol 2004 231 3 357 76 10.1016/j.jtbi.2004.06.031 15501468

[B10] Manca C Tsenova L Bergtold A Freeman S Tovey M Musser JM Barry CE Freedman VH Kaplan G Virulence of a Mycobacterium tuberculosis clinical isolate in mice is determined by failure to induce Th1 type immunity and is associated with induction of IFN-alpha/beta Proc Natl Acad Sci USA 2001 98 10 5752 7 10.1073/pnas.091096998 11320211 PMC33285

[B11] Subbian S Tsenova L Yang G O'Brien P Parsons S Peixoto B Taylor L Fallows D Kaplan G Chronic pulmonary cavitary tuberculosis in rabbits: a failed host immune response Open Biology 2011 1 10.1098/rsob.110016 PMC3352086 22645653

[B12] Flynn JL Tsenova L Izzo A Kaplan G Kaufmann SHE, Britton WJ Experimental animal models of tuberculosis Handbook of Tuberculosis 2008 Weinheim: Wiley-VCH Verlag GmbH & Co 389 426

[B13] Reed MB Domenech P Manca C Su H Barczak AK Kreiswirth BN Kaplan G Barry CE A glycolipid of hypervirulent tuberculosis strains that inhibits the innate immune response Nature 2004 431 7004 84 7 10.1038/nature02837 15343336

[B14] Brennan PJ Crick DC The cell-wall core of Mycobacterium tuberculosis in the context of drug discovery Curr Top Med Chem 2007 7 5 475 88 10.2174/156802607780059763 17346193

[B15] Russell DG Mwandumba HC Rhoades EE Mycobacterium and the coat of many lipids J Cell Biol 2002 158 3 421 6 10.1083/jcb.200205034 12147678 PMC2173834

[B16] Sinsimer D Huet G Manca C Tsenova L Koo MS Kurepina N Kana B Mathema B Marras SA Kreiswirth BN Guilhot C Kaplan G The phenolic glycolipid of Mycobacterium tuberculosis differentially modulates the early host cytokine response but does not in itself confer hypervirulence Infect Immun 2008 76 7 3027 36 10.1128/IAI.01663-07 18443098 PMC2446685

[B17] Kim MJ Wainwright HC Locketz M Bekker LG Walther GB Dittrich C Visser A Wang W Hsu FF Wiehart U Tsenova L Kaplan G Russell DG Caseation of human tuberculosis granulomas correlates with elevated host lipid metabolism EMBO Mol Med 2009 2 7 258 74 10.1002/emmm.201000079 PMC2913288 20597103

[B18] Koo MS Manca C Yang G O'Brien P Sung N Tsenova L Subbian S Fallows D Muller G Ehrt S Kaplan G Phosphodiesterase 4 inhibition reduces innate immunity and improves isoniazid clearance of Mycobacterium tuberculosis in the lungs of infected mice PLoS One 2011 6 2 e17091 10.1371/journal.pone.0017091 21364878 PMC3045423

[B19] Homolka SNS Russell DG Rohde KH Functional genetic diversity among Mycobacterium tuberculosis complex clinical isolates: delineation of conserved core and lineage-specific transcriptomes during intracellular survival PLoS Pathog 2010 6 7 e1000988 10.1371/journal.ppat.1000988 20628579 PMC2900310

[B20] Rohde KH Abramovitch RB Russell DG Mycobacterium tuberculosis invasion of macrophages: linking bacterial gene expression to environmental cues Cell Host Microbe 2007 2 5 352 64 10.1016/j.chom.2007.09.006 18005756

[B21] Subbian S Tsenova L O'Brien P Yang G Koo MS Peixoto B Fallows D Dartois V Muller G Kaplan G Phosphodiesterase-4 Inhibition Alters Gene Expression and Improves Isoniazid - Mediated Clearance of Mycobacterium tuberculosis in Rabbit Lungs PLoS Pathog 2011 7 9 e1002262 10.1371/journal.ppat.1002262 21949656 PMC3174258

[B22] Munoz-Elias EJ Timm J Botha T Chan WT Gomez JE McKinney JD Replication dynamics of Mycobacterium tuberculosis in chronically infected mice Infect Immun 2005 73 1 546 51 10.1128/IAI.73.1.546-551.2005 15618194 PMC538940

[B23] Munoz-Elias EJ McKinney JD Carbon metabolism of intracellular bacteria Cell Microbiol 2006 8 1 10 22 10.1111/j.1462-5822.2005.00648.x 16367862

[B24] Rao V Gao F Chen B Jacobs WR Jr Glickman MS Trans-cyclopropanation of mycolic acids on trehalose dimycolate suppresses Mycobacterium tuberculosis -induced inflammation and virulence J Clin Invest 2006 116 6 1660 7 10.1172/JCI27335 16741578 PMC1464906

[B25] Manca C Tsenova L Barry CE Bergtold A Freeman S Haslett PA Musser JM Freedman VH Kaplan G Mycobacterium tuberculosis CDC1551 induces a more vigorous host response in vivo and in vitro, but is not more virulent than other clinical isolates J Immunol 1999 162 11 6740 6 10352293

[B26] Takeda K Akira S STAT family of transcription factors in cytokine-mediated biological responses Cytokine Growth Factor Rev 2000 11 3 199 207 10.1016/S1359-6101(00)00005-8 10817963

[B27] Schneider A Reichart U Gerner W Kolbe T Saalmuller A Muller M Selective contribution of Tyk2 to cell activation by lipopolysaccharide FEBS Lett 2008 582 25-26 3681 6 10.1016/j.febslet.2008.09.053 18845149

[B28] Karaghiosoff M Neubauer H Lassnig C Kovarik P Schindler H Pircher H McCoy B Bogdan C Decker T Brem G Pfeffer K Muller M Partial impairment of cytokine responses in Tyk2-deficient mice Immunity 2000 13 4 549 60 10.1016/S1074-7613(00)00054-6 11070173

[B29] Zhu L Li H Tang J Zhu J Zhang Y Hyperoxia arrests alveolar development through suppression of histone deacetylases in neonatal rats Pediatr Pulmonol 2011 10.1002/ppul.21540 21905265

[B30] Puxeddu E Knauf JA Sartor MA Mitsutake N Smith EP Medvedovic M Tomlinson CR Moretti S Fagin JA RET/PTC-induced gene expression in thyroid PCCL3 cells reveals early activation of genes involved in regulation of the immune response Endocr Relat Cancer 2005 12 2 319 34 10.1677/erc.1.00947 15947106

[B31] Andresen EGG Bullwinkel J Lange C Heine H Increased expression of beta-defensin 1 (DEFB1) in chronic obstructive pulmonary disease PLoS One 2011 6 7 e21898 10.1371/journal.pone.0021898 21818276 PMC3139569

[B32] Okigaki M Davis C Falasca M Harroch S Felsenfeld DP Sheetz MP Schlessinger J Pyk2 regulates multiple signaling events crucial for macrophage morphology and migration Proc Natl Acad Sci USA 2003 100 19 10740 5 10.1073/pnas.1834348100 12960403 PMC196873

[B33] Hruz P Dann SM Eckmann L STAT3 and its activators in intestinal defense and mucosal homeostasis Curr Opin Gastroenterol 2010 26 2 109 15 10.1097/MOG.0b013e3283365279 20040863

[B34] Hirano T Ishihara K Hibi M Roles of STAT3 in mediating the cell growth, differentiation and survival signals relayed through the IL-6 family of cytokine receptors Oncogene 2000 19 21 2548 56 10.1038/sj.onc.1203551 10851053

[B35] Painz R Walter I Kolbe T Rigler D Vogl C Steinborn R Rulicke T Helmreich M Karaghiosoff M Muller M Organ-specific and differential requirement of TYK2 and IFNAR1 for LPS-induced iNOS expression in vivo Immunobiology 2007 212 9-10 863 75 18086385 10.1016/j.imbio.2007.09.017

[B36] Katsume A Okigaki M Matsui A Che J Adachi Y Kishita E Yamaguchi S Ikeda K Ueyama T Matoba S Yamada H Matsubara H Early inflammatory reactions in atherosclerosis are induced by proline-rich tyrosine kinase/reactive oxygen species-mediated release of tumor necrosis factor-alpha and subsequent activation of the p21Cip1/Ets-1/p300 system Arterioscler Thromb Vasc Biol 2011 31 5 1084 92 10.1161/ATVBAHA.110.221804 21372295

[B37] O'Brien L Carmichael J Lowrie DB Andrew PW Strains of Mycobacterium tuberculosis differ in susceptibility to reactive nitrogen intermediates in vitro Infect Immun 1994 62 11 5187 90 7927804 10.1128/iai.62.11.5187-5190.1994 PMC303246

[B38] Messmer UK Reimer DM Reed JC Brune B Nitric oxide induced poly(ADP-ribose) polymerase cleavage in RAW 264.7 macrophage apoptosis is blocked by Bcl-2 FEBS Lett 1996 384 2 162 6 10.1016/0014-5793(96)00311-0 8612815

[B39] Geijtenbeek TB Kwon DS Torensma R van Vliet SJ van Duijnhoven GC Middel J Cornelissen IL Nottet HS KewalRamani VN Littman DR Figdor CG van Kooyk Y DC-SIGN, a dendritic cell-specific HIV-1-binding protein that enhances trans-infection of T cells Cell 2000 100 5 587 97 10.1016/S0092-8674(00)80694-7 10721995

[B40] Geijtenbeek TB Van Vliet SJ Koppel EA Sanchez-Hernandez M Vandenbroucke-Grauls CM Appelmelk B van Kooyk Y Mycobacteria target DC-SIGN to suppress dendritic cell function J Exp Med 2003 197 1 7 17 12515809 10.1084/jem.20021229 PMC2193797

[B41] Gringhuis SI den Dunnen J Litjens M van Het Hof B van Kooyk Y Geijtenbeek TB C-type lectin DC-SIGN modulates Toll-like receptor signaling via Raf-1 kinase-dependent acetylation of transcription factor NF-kappaB Immunity 2007 26 5 605 16 10.1016/j.immuni.2007.03.012 17462920

[B42] Portevin D Gagneux S Comas I Young D Human macrophage responses to clinical isolates from the Mycobacterium tuberculosis complex discriminate between ancient and modern lineages PLoS Pathog 2011 7 3 e1001307 10.1371/journal.ppat.1001307 21408618 PMC3048359

[B43] Russell DG Cardona PJ Kim MJ Allain S Altare F Foamy macrophages and the progression of the human tuberculosis granuloma Nat Immunol 2009 10 9 943 8 10.1038/ni.1781 19692995 PMC2759071

[B44] Milovanovic I Vujanic M Klun I Bobic B Nikolic A Ivovic V Trbovich AM Djurkovic-Djakovic O Toxoplasma gondii infection induces lipid metabolism alterations in the murine host Mem Inst Oswaldo Cruz 2009 104 2 175 8 19430640 10.1590/s0074-02762009000200008

[B45] Saraswathi V Hasty AH Inhibition of long-chain acyl coenzyme A synthetases during fatty acid loading induces lipotoxicity in macrophages Arterioscler Thromb Vasc Biol 2009 29 11 1937 43 10.1161/ATVBAHA.109.195362 19679826 PMC2766024

[B46] Takano T Tsukiyama-Kohara K Hayashi M Hirata Y Satoh M Tokunaga Y Tateno C Hayashi Y Hishima T Funata N Sudoh M Kohara M Augmentation of DHCR24 expression by hepatitis C virus infection facilitates viral replication in hepatocytes J Hepatol 2011 55 3 512 21 10.1016/j.jhep.2010.12.011 21184787

[B47] Chang KO Role of cholesterol pathways in norovirus replication J Virol 2009 83 17 8587 95 10.1128/JVI.00005-09 19515767 PMC2738148

[B48] Gatfield J Pieters J Essential role for cholesterol in entry of mycobacteria into macrophages Science 2000 288 5471 1647 50 10.1126/science.288.5471.1647 10834844

[B49] Pandey AK Sassetti CM Mycobacterial persistence requires the utilization of host cholesterol Proc Natl Acad Sci USA 2008 105 11 4376 80 10.1073/pnas.0711159105 18334639 PMC2393810

[B50] de Chastellier C The many niches and strategies used by pathogenic mycobacteria for survival within host macrophages Immunobiology 2009 214 7 526 42 10.1016/j.imbio.2008.12.005 19261352

[B51] Schultz RM Pavlidis NA Stylos WA Chirigos MA Regulation of macrophage tumoricidal function: a role for prostaglandins of the E series Science 1978 202 4365 320 1 10.1126/science.694537 694537

[B52] Snyder DS Beller DI Unanue ER Prostaglandins modulate macrophage Ia expression Nature 1982 299 5879 163 5 10.1038/299163a0 6287286

[B53] Rangel Moreno J Estrada Garcia I De La Luz Garcia Hernandez M Aguilar Leon D Marquez R Hernandez Pando R The role of prostaglandin E2 in the immunopathogenesis of experimental pulmonary tuberculosis Immunology 2002 106 2 257 66 10.1046/j.1365-2567.2002.01403.x 12047755 PMC1782721

[B54] Huynh KK Joshi SA Brown EJ A delicate dance: host response to mycobacteria Curr Opin Immunol 2011 23 4 464 72 10.1016/j.coi.2011.06.002 21726990

[B55] Cooper AM Mayer-Barber KD Sher A Role of innate cytokines in mycobacterial infection Mucosal Immunol 2011 4 3 252 60 10.1038/mi.2011.13 21430655 PMC3294290

[B56] Voskuil MI Bartek IL Visconti K Schoolnik GK The response of mycobacterium tuberculosis to reactive oxygen and nitrogen species Front Microbiol 2011 2 105 21734908 10.3389/fmicb.2011.00105 PMC3119406

[B57] Stokes RW Waddell SJ Adjusting to a new home: Mycobacterium tuberculosis gene expression in response to an intracellular lifestyle Future Microbiol 2009 4 10 1317 35 10.2217/fmb.09.94 19995191

[B58] Warner DF Mizrahi V Tuberculosis chemotherapy: the influence of bacillary stress and damage response pathways on drug efficacy Clin Microbiol Rev 2006 19 3 558 70 10.1128/CMR.00060-05 16847086 PMC1539104

[B59] Sherman DR Voskuil M Schnappinger D Liao R Harrell MI Schoolnik GK Regulation of the Mycobacterium tuberculosis hypoxic response gene encoding alpha -crystallin Proc Natl Acad Sci USA 2001 98 13 7534 9 10.1073/pnas.121172498 11416222 PMC34703

[B60] Rustad TR Harrell MI Liao R Sherman DR The enduring hypoxic response of Mycobacterium tuberculosis PLoS One 2008 3 1 e1502 10.1371/journal.pone.0001502 18231589 PMC2198943

[B61] Park HD Guinn KM Harrell MI Liao R Voskuil MI Tompa M Schoolnik GK Sherman DR Rv3133c/dosR is a transcription factor that mediates the hypoxic response of Mycobacterium tuberculosis Mol Microbiol 2003 48 3 833 43 10.1046/j.1365-2958.2003.03474.x 12694625 PMC1992516

[B62] Desjardin LE Hayes LG Sohaskey CD Wayne LG Eisenach KD Microaerophilic induction of the alpha-crystallin chaperone protein homologue (hspX) mRNA of Mycobacterium tuberculosis J Bacteriol 2001 183 18 5311 6 10.1128/JB.183.18.5311-5316.2001 11514514 PMC95413

[B63] Sohaskey CD Wayne LG Role of narK2X and narGHJI in hypoxic upregulation of nitrate reduction by Mycobacterium tuberculosis J Bacteriol 2003 185 24 7247 56 10.1128/JB.185.24.7247-7256.2003 14645286 PMC296237

[B64] Hutter B Dick T Up-regulation of narX, encoding a putative 'fused nitrate reductase' in anaerobic dormant Mycobacterium bovis BCG FEMS Microbiol Lett 1999 178 1 63 9 10.1111/j.1574-6968.1999.tb13760.x 10483724

[B65] Converse PJ Karakousis PC Klinkenberg LG Kesavan AK Ly LH Allen SS Grosset JH Jain SK Lamichhane G Manabe YC McMurray DN Nuermberger EL Bishai WR Role of the dosR-dosS two-component regulatory system in Mycobacterium tuberculosis virulence in three animal models Infect Immun 2009 77 3 1230 7 10.1128/IAI.01117-08 19103767 PMC2643651

[B66] Glickman MS Cahill SM Jacobs WR Jr The Mycobacterium tuberculosis cmaA2 gene encodes a mycolic acid trans-cyclopropane synthetase J Biol Chem 2001 276 3 2228 33 10.1074/jbc.C000652200 11092877

[B67] Rao V Fujiwara N Porcelli SA Glickman MS Mycobacterium tuberculosis controls host innate immune activation through cyclopropane modification of a glycolipid effector molecule J Exp Med 2005 201 4 535 43 10.1084/jem.20041668 15710652 PMC2213067

[B68] Vandal OH Pierini LM Schnappinger D Nathan CF Ehrt S A membrane protein preserves intrabacterial pH in intraphagosomal Mycobacterium tuberculosis Nat Med 2008 14 8 849 54 10.1038/nm.1795 18641659 PMC2538620

[B69] Biswas T Small J Vandal O Odaira T Deng H Ehrt S Tsodikov OV Structural insight into serine protease Rv3671c that Protects M. tuberculosis from oxidative and acidic stress Structure 2011 18 10 1353 63 10.1016/j.str.2010.06.017 PMC2955984 20947023

[B70] McKinney JD Honer zu Bentrup K Munoz-Elias EJ Miczak A Chen B Chan WT Swenson D Sacchettini JC Jacobs WR Jr Russell DG Persistence of Mycobacterium tuberculosis in macrophages and mice requires the glyoxylate shunt enzyme isocitrate lyase Nature 2000 406 6797 735 8 10.1038/35021074 10963599

[B71] Schnappinger D Ehrt S Voskuil MI Liu Y Mangan JA Monahan IM Dolganov G Efron B Butcher PD Nathan C Schoolnik GK Transcriptional Adaptation of Mycobacterium tuberculosis within Macrophages: Insights into the Phagosomal Environment J Exp Med 2003 198 5 693 704 10.1084/jem.20030846 12953091 PMC2194186

[B72] Timm J Post FA Bekker LG Walther GB Wainwright HC Manganelli R Chan WT Tsenova L Gold B Smith I Kaplan G McKinney JD Differential expression of iron-, carbon-, and oxygen-responsive mycobacterial genes in the lungs of chronically infected mice and tuberculosis patients Proc Natl Acad Sci USA 2003 100 24 14321 6 10.1073/pnas.2436197100 14623960 PMC283590

[B73] Ehrt S Schnappinger D Bekiranov S Drenkow J Shi S Gingeras TR Gaasterland T Schoolnik G Nathan C Reprogramming of the macrophage transcriptome in response to interferon-gamma and Mycobacterium tuberculosis: signaling roles of nitric oxide synthase-2 and phagocyte oxidase J Exp Med 2001 194 8 1123 40 10.1084/jem.194.8.1123 11602641 PMC2193509

[B74] Subbian S Mehta PK Cirillo SL Bermudez LE Cirillo JD A Mycobacterium marinum mel2 mutant is defective for growth in macrophages that produce reactive oxygen and reactive nitrogen species Infect Immun 2007 75 1 127 34 10.1128/IAI.01000-06 17030568 PMC1828420

