# Supplementary material for: Strain specific transcriptional response in Mycobacterium tuberculosis infected macrophages
Source: Cell Commun Signal. 2012 Jan 26;10:2. doi: 10.1186/1478-811X-10-2 (PMC3317440; doi:10.1186/1478-811X-10-2)
Supplement: Additional file 1 — Top ten differentially expressed host genes by CDC1551 and HN878 infection at 6 and 24 hours. The top 10 differentially expressed macrophage genes during infection by CDC1551 or HN878 at 6 and 24 hours were grouped according to their level of expression and significance (P value). The macrophage genes commonly up-regulated at 6 hpi between CDC1551 and HN878 are highlighted in red; commonly down-regulated at 6 hpi are marked in green; commonly up-regulated at 24 hpi are shown in purple color and commonly down-regulated at 24 hpi are denoted in blue. [file 1478-811X-10-2-S1.DOC]

**Additional File 1- Top ten differentially expressed host genes during infection by CDC1551 and HN878 at 6 and 24 hours**

| **Up-regulated** | | | |  | **Down-regulated** | | | | | | |
| --- | --- | --- | --- | --- | --- | --- | --- | --- | --- | --- | --- |
| **ID** | **Gene** | ***P* value** | **Fold change** |  | **ID** | | **Gene** | | ***P* value** | | **Fold change** |
|  |  |  |  |  |  |  | |  | |  | |
| **CDC1551_6h** |  |  |  |  |  |  | |  | |  | |
| NM_011198 | **Ptgs2** | 8.48E-20 | 192.83 |  | NM_001042591 | **Arrdc3** | | 2.32E-19 | | -24.32 | |
| NM_008392 | **Irg1** | 6.03E-22 | 159.35 |  | NM_175116 | **P2ry5** | | 3.38E-18 | | -16.46 | |
| NM_031168 | **Il6** | 2.84E-15 | 142.97 |  | NM_176837 | **Arhgap18** | | 1.73E-19 | | -15.97 | |
| NM_172648 | **Ifi205** | 1.25E-14 | 138.35 |  | NM_011994 | **Abcd2** | | 4.96E-16 | | -15.22 | |
| NM_010104 | **Edn1** | 5.78E-17 | 109.95 |  | NM_207246 | **Rasgrp3** | | 3.16E-17 | | -15.15 | |
| NM_001033122 | **Cd69** | 7.27E-15 | 108.48 |  | NM_145933 | **St6gal1** | | 8.88E-17 | | -13.31 | |
| NM_008599 | **Cxcl9** | 1.53E-22 | 100.45 |  | NM_011050 | **Pdcd4** | | 9.29E-14 | | -12.75 | |
| NM_010554 | **Il1α** | 3.61E-14 | 85.38 |  | NM_008278 | **Hpgd** | | 1.75E-14 | | -12.13 | |
| NM_010927 | **Nos2** | 1.18E-15 | 78.41 |  | NM_027872 | **Slc46a3** | | 1.14E-16 | | -11.69 | |
| NM_011331 | **Ccl12** | 7.73E-15 | 77.21 |  | NM_015786 | **Hist1h1c** | | 7.29E-14 | | -11.65 | |
|  |  |  |  |  |  |  | |  | |  | |
| **HN878_6h** |  |  |  |  |  |  | |  | |  | |
| NM_008392 | **Irg1** | 6.30E-22 | 157.62 |  | NM_008278 | **Hpgd** | | 2.91E-16 | | -21.95 | |
| NM_011198 | **Ptgs2** | 2.69E-19 | 143.00 |  | NM_001042591 | **Arrdc3** | | 7.69E-19 | | -20.14 | |
| NM_031168 | **Il6** | 4.98E-15 | 123.90 |  | NM_001042591 | **Rasgrp3** | | 1.96E-17 | | -16.20 | |
| NM_172648 | **Ifi205** | 3.25E-14 | 108.58 |  | NM_207246 | **Abcd2** | | 6.17E-16 | | -14.76 | |
| NM_010554 | **Il1α** | 1.72E-14 | 101.96 |  | NM_011994 | **P2ry5** | | 8.96E-18 | | -14.37 | |
| NM_001033122 | **Cd69** | 1.77E-14 | 87.58 |  | NM_175116 | **Arhgap18** | | 6.61E-19 | | -13.31 | |
| NM_009140 | **Cxcl2** | 6.33E-18 | 84.89 |  | NM_176837 | **Gpr155** | | 7.18E-18 | | -12.82 | |
| NM_011315 | **Saa3** | 8.21E-22 | 76.25 |  | NM_001080707 | **BC031353** | | 9.08E-18 | | -11.87 | |
| NM_008361 | **Il1β** | 2.07E-16 | 64.09 |  | NM_001113283 | **St6gal1** | | 3.11E-16 | | -11.32 | |
| NM_013521 | **Fpr1** | 2.86E-17 | 62.60 |  | NM_145933 | **Cnr2** | | 1.78E-18 | | -10.75 | |
|  |  |  |  |  |  |  | |  | |  | |
| **CDC1551_24h** |  |  |  |  |  |  | |  | |  | |
| NM_011315 | **Saa3** | 2.98E-23 | 167.72 |  | NM_008278 | **Hpgd** | | 1.67E-18 | | -55.93 | |
| NM_008491 | **Lcn2** | 9.06E-17 | 120.51 |  | NM_009888 | **Cfh** | | 3.36E-13 | | -26.57 | |
| NM_007646 | **Cd38** | 1.10E-14 | 55.05 |  | NM_028784 | **F13a1** | | 4.58E-13 | | -23.49 | |
| NM_008392 | **Irg1** | 8.37E-20 | 52.02 |  | NM_010796 | **Mgl1** | | 1.42E-14 | | -20.71 | |
| NM_054055 | **Slc13a3** | 3.48E-17 | 46.40 |  | NM_020008 | **Clec7a** | | 8.31E-18 | | -17.35 | |
| NM_010766 | **Marco** | 7.41E-18 | 40.99 |  | NM_008625 | **Mrc1** | | 1.25E-16 | | -16.85 | |
| NM_013521 | **Fpr1** | 6.96E-16 | 33.43 |  | NM_026162 | **Plxdc2** | | 7.09E-14 | | -15.09 | |
| NM_008599 | **Cxcl9** | 2.52E-19 | 23.79 |  | NM_028072 | **Sulf2** | | 5.62E-20 | | -14.44 | |
| NM_008198 | **Cfb** | 6.31E-13 | 20.01 |  | NM_139200 | **Cytip** | | 1.63E-17 | | -14.09 | |
| NM_007695 | **Chi3l1** | 1.94E-13 | 18.23 |  | NM_010145 | **Ephx1** | | 5.12E-15 | | -13.85 | |
|  |  |  |  |  |  |  | |  | |  | |
| **HN878_24h** |  |  |  |  |  |  | |  | |  | |
| NM_011315 | **Saa3** | 2.66E-23 | 172.62 |  | NM_008278 | **Hpgd** | | 8.45E-18 | | -40.62 | |
| NM_008491 | **Lcn2** | 3.93E-16 | 84.97 |  | NM_009888 | **Cfh** | | 1.39E-12 | | -20.88 | |
| NM_007646 | **Cd38** | 2.98E-15 | 73.07 |  | NM_010796 | **Mgl1** | | 2.95E-14 | | -18.47 | |
| NM_008392 | **Irg1** | 2.33E-20 | 67.79 |  | NM_028784 | **F13a1** | | 4.00E-12 | | -16.58 | |
| BC038121 | **Cd209f** | 3.49E-13 | 65.09 |  | NM_008625 | **Mrc1** | | 1.84E-16 | | -15.94 | |
| NM_054055 | **Slc13a3** | 1.52E-17 | 54.80 |  | NM_146042 | **Rnf144b** | | 4.82E-17 | | -14.55 | |
| NM_010766 | **Marco** | 2.15E-18 | 52.21 |  | NM_175316 | **Slco2b1** | | 2.66E-17 | | -13.65 | |
| NM_013521 | **Fpr1** | 5.33E-16 | 35.10 |  | NM_007642 | **Cd28** | | 1.27E-10 | | -13.53 | |
| NM_010554 | **Il1α** | 1.10E-11 | 26.33 |  | NM_028072 | **Sulf2** | | 1.63E-19 | | -12.55 | |
| NM_011198 | **Ptgs2** | 1.57E-15 | 24.06 |  | NM_007553 | **Bmp2** | | 7.65E-14 | | -12.12 | |
