# Supplementary material for: Strain specific transcriptional response in Mycobacterium tuberculosis infected macrophages
Source: Cell Commun Signal. 2012 Jan 26;10:2. doi: 10.1186/1478-811X-10-2 (PMC3317440; doi:10.1186/1478-811X-10-2)
Supplement: Additional file 3 — Expression levels of macrophage EIAN and lipid metabolism genes during infection by Mtb. The differentially expressed host genes upon BMM infection with CDC1551 or HN878 at 6 and 24 hpi were identified by microarray analysis and subjected to functional pathway analysis using IPA. Two networks, an early immune activation network (EIAN) at 6 hpi and a sub-network of lipid metabolism pathway at 24 hpi, were differentially modulated in CDC1551 or HN878 infected BMM, respectively. The fold changes in the level of expression of genes that constitute those two networks are shown. [file 1478-811X-10-2-S3.DOC]

| Gene | ID | | Gene Assignment | Fold change | |
| --- | --- | --- | --- | --- | --- |
|  |  |  |  | CDC1551 | HN878 |
|  |  | |  |  |  |
| **Early Immune Activation Network (6 hours post-infection)** | | | |  |  |
| *Stat3* | NM_213659 | signal transducer/activator of transcription 3 | | 2.80 | 1.88 |
| *Stat5A* | NM_011488 | signal transducer/activator of transcription 5A | | 2.32 | 1.93 |
| *Atf3* | NM_007498 | activating transcription factor 3 | | 2.38 | 1.89 |
| *Hdac1* | NM_008228 | histone deacetylase 1 | | 2.10 | 1.55 |
| *Ptk2B* | NM_172498 | protein tyrosine kinase 2 beta | | 2.19 | 1.57 |
| *Cish* | NM_009895 | cytokine inducible SH2-containing protein | | 2.00 | 1.89 |
| *Tyk2* | NM_018793 | tyrosine kinase 2 | | 2.51 | 1.73 |
| *Il4R* | NM_001008700 | interleukin 4 receptor | | 2.01 | 1.25 |
| *Il12RB1* | NM_008353 | interleukin 12 receptor B1 | | 2.38 | 1.44 |
| *Notch1* | NM_145431 | notch 1 | | 2.45 | 1.94 |
| *Itga4* | NM_010576 | integrin, alpha 4 (antigen CD49D, α4) | | 2.38 | 1.44 |
| *Ccr7* | NM_007719 | chemokine (C-C motif) receptor 7 | | 2.28 | 1.65 |
|  |  |  | |  |  |
| **Lipid metabolism network (24 hours post-infection)** | | | |  |  |
| *Fasn* | NM_007988 | fatty acid synthase | | 1.69 | 2.09 |
| *Insig1* | NM_153526 | insulin induced gene 1 | | 1.75 | 2.10 |
| *Stard4* | NM_133774 | StAR-related lipid transfer domain containing 4 | | 1.91 | 2.46 |
| *Hmgcr* | NM_008255 | 3-hydroxy-3-methylglutaryl-CoA reductase | | 1.92 | 2.39 |
| *Nsdhl* | NM_010941 | NAD(P) dependent steroid dehydrogenase-like | | 1.88 | 2.34 |
| *Dhcr24* | NM_053272 | 24-dehydrocholesterol reductase | | 1.68 | 2.48 |
| *Dhcr7* | NM_007856 | 7-dehydrocholesterol reductase | | 1.63 | 1.95 |
| *Acss2* | NM_019811 | [acyl-CoA synthetase short-chain family 2](http://www.genecards.org/cgi-bin/carddisp.pl?gene=ACSS2&search=acss2) | | 1.84 | 2.82 |
| *Sc4mol* | NM_025436 | [sterol-C4-methyl oxidase-like1](http://www.genenames.org/data/hgnc_data.php?hgnc_id=10545) | | 1.77 | 2.25 |
| *Gbp5* | NM_153564 | [guanylate binding protein 5](http://www.genecards.org/cgi-bin/carddisp.pl?gene=GBP5&search=gbp5) | | 1.87 | 2.30 |
| *Scd1* | NM_009127 | stearoyl-CoA desaturease 1 | | 1.23 | 2.28 |
| *Tnf* | NM_013693 | tumor necrosis factor | | 3.72 | 4.79 |
| *Pparg* | NM_001127330 | [peroxisome proliferator-activated receptor γ](http://www.genecards.org/cgi-bin/carddisp.pl?gene=PPARG&search=pparg) | | -2.39 | -3.33 |
| *Igf1* | NM_010512/ | insulin growth factor 1 | | -3.01 | -2.60 |
| *CcnD1* | NM_007631 | cycline D1 | | -2.13 | -4.05 |

**Additional File 3. Expression levels of macrophage EIAN and lipid metabolism genes during *Mtb* infection**
