# Supplementary figures and images for: Strain specific transcriptional response in Mycobacterium tuberculosis infected macrophages
Source: Cell Commun Signal. 2012 Jan 26;10:2. doi: 10.1186/1478-811X-10-2 (PMC3317440; doi:10.1186/1478-811X-10-2)

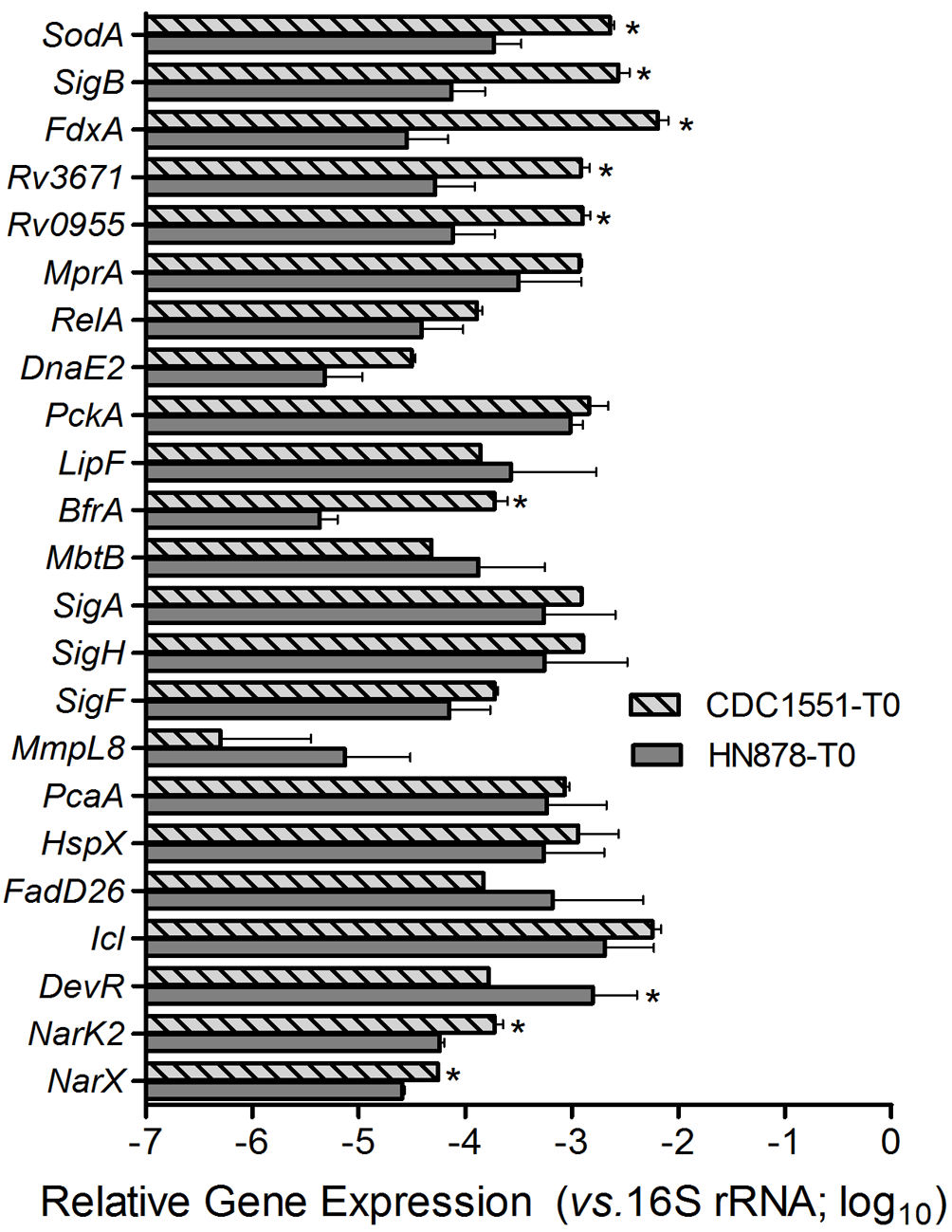

Supplement: Additional file 4 — Basal level expression of mycobacterial genes upon infection of BMDM. Total RNA was isolated from CDC1551 or HN878 after 3 h infection of BMM (T0) and the level of expression of Mtb genes involved in hypoxia, general stress response and fatty acid/lipid metabolism pathways was determined by qRT-PCR. The amount of transcripts for each Mtb gene was normalized to 16S rRNA levels. The results are means ± standard deviation from three independent samples repeated at least twice. * P ≤ 0.05. [file 1478-811X-10-2-S4.TIFF]
